# Transferrin-Bound Doxorubicin Enhances Apoptosis and DNA Damage through the Generation of Pro-Inflammatory Responses in Human Leukemia Cells

**DOI:** 10.3390/ijms21249390

**Published:** 2020-12-10

**Authors:** Monika Jedrzejczyk, Katarzyna Wisniewska, Katarzyna Dominika Kania, Agnieszka Marczak, Marzena Szwed

**Affiliations:** 1 Department of Medical Biophysics, Institute of Biophysics, Faculty of Biology and Environmental Protection, University of Lodz, Pomorska 141/143 Street, 90-236 Lodz, Poland; mon_ag_wer@wp.pl (M.J.); kasiaawisniewska@interia.pl (K.W.); agnieszka.marczak@biol.uni.lodz.pl (A.M.); 2 Laboratory of Transcriptional Regulation, Institute for Medical Biology, PAS, Lodowa 106 Street, 93-232 Lodz, Poland; kkania@cbm.pan.pl

**Keywords:** leukemias, doxorubicin, inflammation

## Abstract

Doxorubicin (DOX) is an effective antineoplastic drug against many solid tumors and hematological malignancies. However, the clinical use of DOX is limited, because of its unspecific mode of action. Since leukemia cells overexpress transferrin (Tf) receptors on their surface, we proposed doxorubicin–transferrin (DOX–Tf) conjugate as a new vehicle to increase drug concentration directly in cancer cells. The data obtained after experiments performed on K562 and CCRF-CEM human leukemia cell lines clearly indicate severe cytotoxic and genotoxic properties of the conjugate drug. On the other hand, normal peripheral blood mononuclear cells (PBMCs) were more resistant to DOX–Tf than to DOX. In comparison to free drug, we observed that Tf-bound DOX induced apoptosis in a TRAIL-dependent manner and caused DNA damage typical of programmed cell death. These fatal hallmarks of cell death were confirmed upon morphological observation of cells incubated with DOX or DOX–Tf. Studies of expression of *TNF-α, IL-4, and IL-6* at the mRNA and protein levels revealed that the pro-inflammatory response plays an important role in the toxicity of the conjugate. Altogether, the results demonstrated here describe a mechanism of the antitumor activity of the DOX–Tf conjugate.

## 1. Introduction

Despite the key achievements obtained with novel biological agents developed during the last decade, leukemia remains an incurable disease [1]. One of the major challenges related to the treatment of leukemia is the huge diversity of these neoplasms [2,3]. The pathogenesis of the most common types of hematological malignancies, acute lymphoblastic leukemia (ALL), and chronic myelogenous leukemia (CML), involves successive genomic alterations in multipotent hematopoietic stem cells, and leads to their abnormal proliferation. Although CML is characterized by the constitutively activated BCR/ABL tyrosine kinase that confers resistance to apoptosis induced by anticancer drugs [4], ALL is typically of unknown etiology. ALL can develop from underlying hematological disorders (e.g., myelodysplastic syndromes) or from exposure to genotoxic agents (e.g., Type II topoisomerases, alkylating agents, or radiation) [5]. Another challenge with chemotherapy for leukemia is the development of treatment resistance [6], and accordingly there is a constant search for new therapeutic targets to improve patient survival rate and quality of life. 

Anthracyclines, including doxorubicin (DOX), are commonly used for the treatment of both solid tumors and hematologic malignancies [7]. DOX exerts its cytotoxicity by producing free radicals, intercalating with DNA base pairs, and interacting with several molecular targets such as DNA topoisomerase II. There are several side effects that have been observed in patients treated with DOX, such as skin irritation, nausea, fever, and severe cardiac toxicity, leading to increased risk of heart failure [8]. Several years ago, it was demonstrated that the DOX-induced pro-inflammatory response may play a crucial role in the symptoms associated with anthracycline therapy [9,10]. DOX triggers inflammatory signaling cascades by promoting the release of different cytokines, including interleukin-1 (IL-1) and tumor necrosis factor-α (TNF-α), in combination with the activation of various signaling pathways, such as nuclear factor-κB (NF-κB), p38-MAPK, and autophagy pathways [11]. Moreover, the cytokines and inflammatory markers released after DOX stimulation are associated with the induction of the extrinsic apoptotic pathway [12,13]. For instance, TNF-related apoptosis-inducing ligand (TRAIL) is highly expressed after various anticancer drug treatments. Upon binding to TNF death receptors, TRAIL leads to their aggregation and recruits Fas-associated death domain-containing protein (FADD). The death domain of FADD binds to an analogous domain of caspase-8 to form the death-inducing signaling complex (DISC). This process activates downstream caspases and leads to programmed cell death [14]. The extrinsic apoptotic pathway may remain functional and successfully kill cancer cells, unless DOX treatment results in nonspecific cytotoxic action towards normal bone marrow, renal, and heart tissue. 

Transferrin receptors (TfR) are overexpressed on many malignant tumor cells [15,16], whereas normal cells are deficient in this type of receptor [17]. Importantly, erythroid precursors, as well as their malignant clones represent those groups of cells that have the highest expression of TfRs [18]. 

Therefore, we suggested that transferrin (Tf) may be used as a carrier for DOX. For this reason, we used two different human leukemia cell lines CCRF-CEM (1 × 10^5^ TfR per cell [19]) and K562 (1.5 × 10^5^ TfR per cell, [20]) that represent ALL and chronic myeloid leukemia, respectively. 

In parallel, the experiments were carried out on normal, non-cancer peripheral blood mononuclear cells (PBMCs) acquired from healthy donors. By conjugating DOX with transferrin (DOX–Tf conjugate), we proposed that Tf-bound DOX would improve drug cytotoxicity toward human cancer cells and that normal cells would remain intact. This hypothesis was partially proven in our previous study, showing that the conjugate was better able to induce programmed cell death compared to free DOX [21]. Moreover, when we compared the activities of different caspases involved in conjugate-mediated induction of apoptosis, we noticed the activation of casapse-8. This may indicate a contribution of the extrinsic apoptotic pathway to cytotoxicity with the conjugate. Therefore, we chose to explore the involvement of cell death receptor machinery as a component of the DOX–Tf-induced pro-death response in human leukemia cells. 

In the current study, we demonstrate that the toxicity of DOX–Tf is much higher than that of free DOX in CCRF-CEM and K562 human leukemia cells in vitro. In addition, the conjugate is less cytotoxic to normal peripheral mononuclear cells than it is to each of the leukemia cell lines. We confirm the selective activity of Tf-bound DOX towards cancer cells by assessing genotoxic properties of the conjugate. Interestingly, our previous studies performed on solid tumor cells as well as hematological malignancies clearly show the predominant properties of DOX–Tf conjugate to induce apoptosis [22,23]. Here we find that pro-inflammatory cytokines participate in the cytotoxic reaction triggered by the conjugate, and this may be associated with the active delivery of a higher DOX dosage to cancer cells via a transferrin-dependent pathway. 

## 2. Results

### 2.1. Various Forms of Doxorubicin Exert Differential Cytotoxicity in Human Leukemia Cells

**Figure 1:**
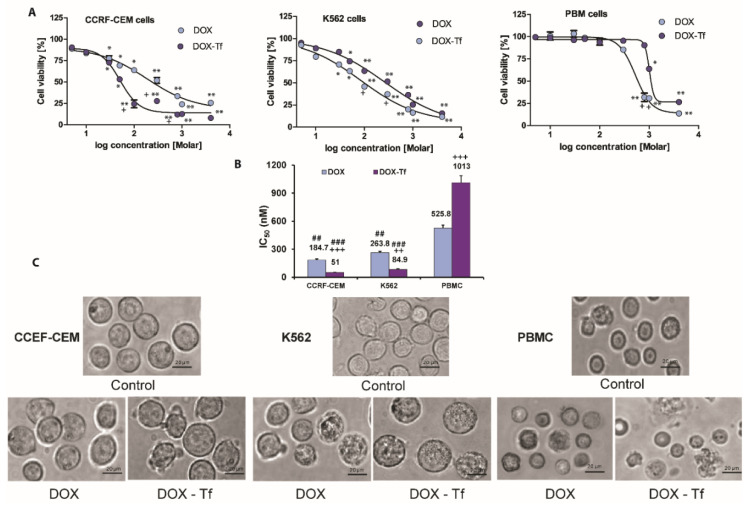
Cytotoxicity of free doxorubicin and doxorubicin–transferrin conjugate in human leukemia cell lines and peripheral blood mononuclear cells. (**A**): Viability of CCRF-CEM, K562, and PBMC cells after 48 h incubation with increasing concentrations of doxorubicin–transferrin (DOX–Tf) conjugate (violet symbols) or free doxorubicin (DOX) (blue symbols). Cell viability was measured by XTT assay. Values represent the means ± SD, (*n* = 5) * *p* < 0.05, ** *p* < 0.01 in comparison to untreated, control cells, (+) statistically significant differences noted between the probes incubated with free DOX compared to the conjugate, + *p* < 0.05. (**B**): Comparison of the cytotoxicity of free DOX and transferrin-bound DOX in CCRF-CEM and K562 cell lines or PBMCs. ^##^ *p* < 0.05, ^###^ *p* < 0.01 in comparison to normal, non-cancer cells, (++, +++) statistically significant differences noted between the probes incubated with free DOX compared to the conjugate, ++ *p* < 0.01, +++ *p* < 0.001. The values are the IC_50_ mean [nM] ± SD of five independent experiments with a 95% confidence interval. (**C**): Morphological changes observed with microscopy. Inverted phase contrast microscopy images were obtained following treatment of CCRF-CEM and K562 cells or PBMCs for 48 h with DOX–Tf or free DOX with the IC_50_ concentrations shown in the photos. Images were captured at 20× magnification, and the scale bars represent 20 µm.

To assess the cytotoxicity of Tf-bound DOX and free drug in human leukemia cells, we measured the mitochondrial activity of respiratory chain oxidoreductases that are active only in living cells. Reduced cell viability was observed in CCRF-CEM and K562 cell lines following DOX treatment (Figure 1A), and this was further enhanced in cells treated with the conjugate. In contrast, free DOX was more cytotoxic to PBMCs than the conjugate. This agrees with our previous finding showing that both forms of DOX displayed diverse cytotoxicity in solid tumor cell lines, as determined by the varied panel of viability assays. As shown in Figure 1B, K562 and CCRF-CEM leukemia cell lines were consistently more sensitive to DOX–Tf than to DOX. In contrast, normal PBMCs were 2-fold less sensitive to DOX–Tf conjugate than to free drug, and this difference was significant. This observation is supported by microscopy studies (Figure 1C) showing that DOX–Tf affects the cellular morphology and induces morphological changes such as cell shrinkage and membrane protrusions.

### 2.2. DOX–Tf Conjugate Generates the Accumulation of ɣH2AX Phosphorylation

**Figure 2:**
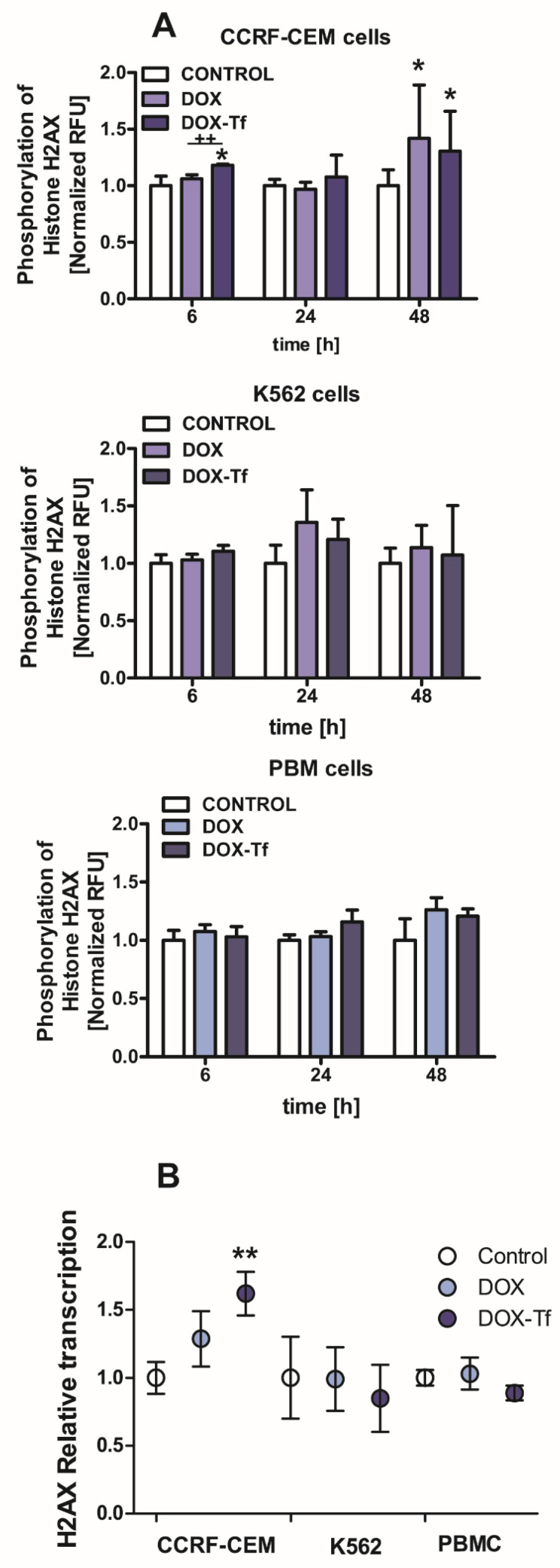
Doxorubicin–transferrin conjugate induced modifications of histone H2AX in human leukemia cells (**A**):The ratio of phosphorylation of histone H2AX (γH2AX) in comparison to total cellular content of this protein after treatment of CCRF-CEM and K562 cells or PBMCs with IC_50_ concentrations of doxorubicin (DOX) alone and doxorubicin–transferrin (DOX–Tf) conjugate for 6, 24, or 48 h. All values were normalized to untreated control cells, taken as 1. Data are expressed as the means ± SD, (*n* = 3). * *p* < 0.05 indicates statistically significant difference compared with control cells; and ++ *p* < 0.01 shows a difference of γH2AX level between cells treated with DOX or DOX–Tf. (**B**): The level of mRNA transcripts for the histone *H2AX* gene in the examined human leukemia cell lines as well as PBMCs exposed to IC_50_ concentrations of free DOX or DOX–Tf for 24 h. Data are expressed as the means ± SD, (*n* = 3). Asterisks refer to the level of significant difference (** *p* < 0.01) in mRNA level in the conjugate-treated cells compared to untreated control cells.

The reduction in cell viability triggered by the conjugate may be related to the various features of DOX–Tf toxicity, such as genotoxicity. Therefore, we measured the phosphorylation of histone H2AX, which is a molecular marker of dsDNA breaks. Our previous findings showed that Tf-bound DOX significantly induced DNA damage in both solid tumor and leukemia cell lines [24], demonstrating that the conjugate caused DNA lesions and the formation of alkali-labile sites. Here, we aimed to determine whether DOX–Tf triggered dsDNA breaks in two malignant cell lines, versus noncancerous PBMCs. As shown in Figure 2A, we found a significant increase in phosphorylation of histone H2A, predominantly in CCRF-CEM cells after 6 and 48 h of drug treatment. Under the same conditions, we observed a predominant role of the conjugate that induced 1.2- and 1.4-fold increases in intracellular ɣH2AX levels. In contrast, 1.3-fold growth was elicited by free DOX in K562 cells after a 24 h incubation. Furthermore, mostly in the CCRF-CEM cell line, DOX–Tf conjugate treatment led to an increase in histone *H2AX* transcription as the first cellular response to DNA lesions (Figure 2B).

### 2.3. Conjugate-Dependent DNA Damage/Lesions Are Connected to Apoptotic Cell Death

Intrigued by the increasing level of histone H2AX, we further analyzed whether the DNA damage induced by DOX was the molecular consequence of activated programmed cell death pathways. With transferase dUTP nick end labeling (TUNEL) assay, we measured pro-apoptotic DNA fragmentation to estimate the fraction of cells that exhibited single- and dsDNA fragments with possible label-free 3’-OH ends following treatment with DOX or DOX–Tf conjugate. 

**Figure 3:**
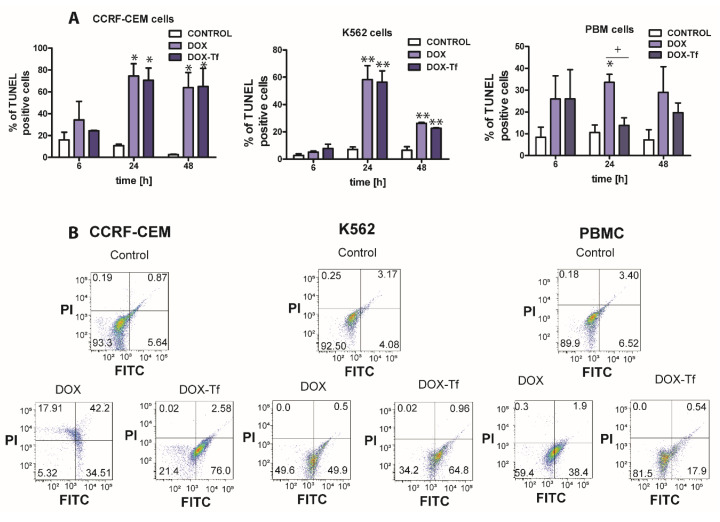
DNA damage is correlated with doxorubicin–transferrin-induced apoptosis in CCRF-CEM and K562 cell lines and in PBMCs (**A**) The influence free doxorubicin (DOX) and transferrin (Tf)-bound DOX (IC_50_ concentrations, respectively) on the induction of apoptosis in human leukemia cells as well as normal PBMCs was determined by transferase dUTP nick end labeling (TUNEL) assay. Quantitative results, calculated as the level of TUNEL-positive cells, are presented as mean ± SD (≥3). * *p* < 0.05, ** *p* < 0.01 relative to the control (untreated cells); + *p* < 0.05 indicates statistically significant difference between cells treated with DOX or DOX–Tf. (**B**) Typical cytometric dot-blots obtained after 24 h of incubation of human cancer and normal cells with IC_50_ concentrations of DOX alone or DOX–Tf conjugate. Lower left down corner, TUNEL-negative cells, propidium iodide (PI)-negative; lower right corner, TUNEL-positive cells, PI-negative; left upper corner, TUNEL-negative, PI-positive cells; upper right corner, TUNEL-positive, PI-positive cells.

As shown in Figure 3A,B, the population of TUNEL-positive cells increased significantly when treated with free or conjugated DOX. The presence of DNA fragments with possible label-free 3’-OH ends was the highest in CCRF-CEM cells, and apoptosis increased from 20% to approximately 75% after 6 and 24 h of incubation with free or conjugated DOX. However, upon analysis of TUNEL-positive cells in the PBMC culture, it was revealed that DOX alone markedly increased the population of cells displaying DNA fragmentation, which is typical of apoptotic cell death.

### 2.4. The Extrinsic TRAIL-Dependent Apoptotic Pathway Is Triggered by DOX–Tf Conjugate in Human Leukemia Cells 

**Figure 4:**
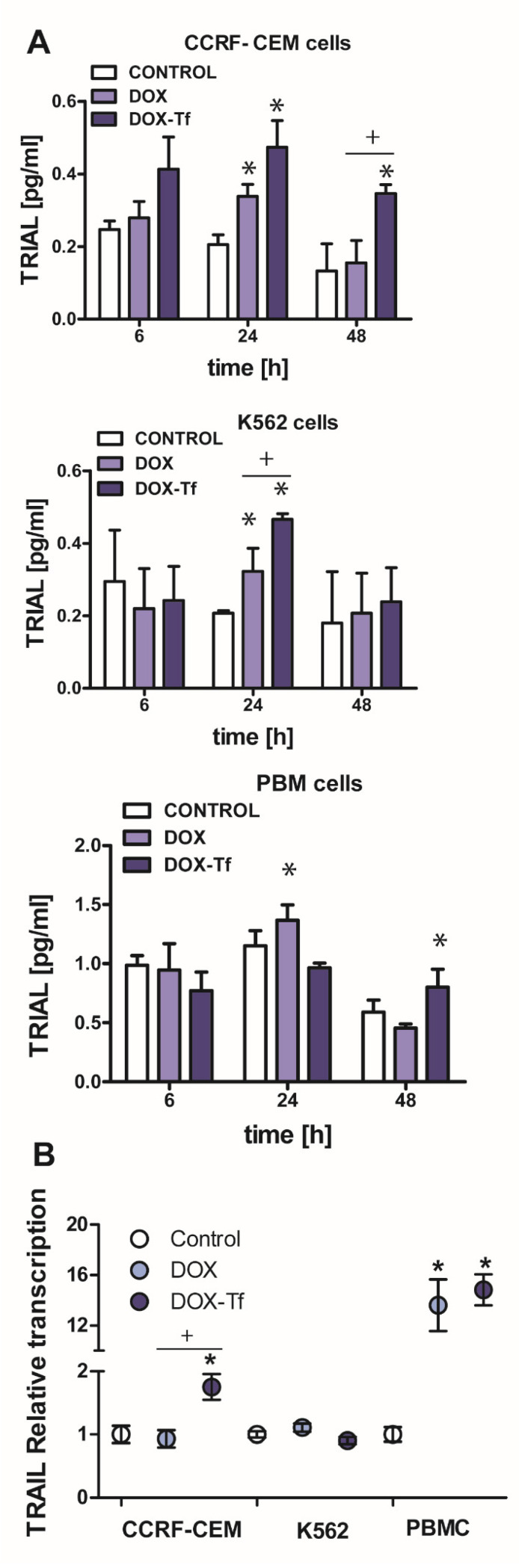
Doxorubicin -transferrin conjugate alters TRAIL ligand expression in human leukemia cells. (**A**) The production of TRAIL-ligand (TRAIL-L) in cancer and normal cells incubated with IC_50_ concentrations of doxorubicin (DOX) and doxorubicin–transferrin (DOX–Tf) conjugate for 6, 24, or 48 h. The results represent mean ± SD of three independent experiments. * *p* < 0.05 in comparison to respective control cells taken as 100%, (+) statistically significant difference observed between cells incubated with DOX in comparison to DOX–Tf, + *p* < 0.05. (**B**) *TRAIL-L* transcript levels (relative to *Hydroxymethylbilane synthase (HPRT1)* and *hydroxymethylbilane synthase (HMBS)* housekeeping genes) in CCRF-CEM and K562 cell lines and PBMCs exposed to either form of DOX for 24 h at IC_50_ concentrations, *n* = 3. Asterisks refer to significant differences (* *p* < 0.05) in the transcription levels in cells treated with free drug or DOX–Tf compared to the untreated cells.  + indicates statistically significant changes between samples incubated with DOX alone or with DOX–Tf (+ *p*  <  0.05).

Based on our previous data that the conjugate causes a reduction of mitochondrial membrane potential, provokes cytochrome c leakage, and mediates the activation of caspase-3 [21,23] we next asked the question whether the extrinsic pathway of apoptosis is induced by DOX–Tf treatment in human leukemia cells. In addition to our previous findings, this hypothesis was developed based on caspase-8 activity measurements that revealed the possibility that the conjugate induced a TRIAL-dependent [21] mechanism of cell death. Indeed, an increase in TRAIL activity was observed in both leukemia cell lines after 24 and 48 h incubation with DOX or DOX–Tf. As shown in Figure 4A, we observed 2.6- and 1.4-fold higher TRAIL expression between samples incubated with the conjugate or with free DOX in CCRF-CEM and K562 cells, respectively. In contrast, the increase in TRAIL ligand (TRAIL-L) levels in normal PBMC cultures was induced only after 24 h continuous incubation with free DOX. To determine whether the effect of the conjugate on TRAIL ligand production was attributed to changes at the mRNA level, we decided to measure *TRIAL–L* gene transcript. Figure 4B shows that both forms of DOX stimulated *TRAIL-L* expression in PBMCs, which suggests a substantial influence of free drug and the conjugate to induce of the extrinsic apoptotic pathway in human leukemia cells. However, a statistically significant difference in *TRAIL-L* mRNA level was observed between DOX-treated and conjugate-treated samples only in CCRF-CEM cell lines, confirming a superior effect of the conjugate in acute leukemia cells. 

### 2.5. The Growth of TNF-α Expression Is Initiated by Tf-Bound DOX and Free Drug in Human Leukemia Cells

**Figure 5:**
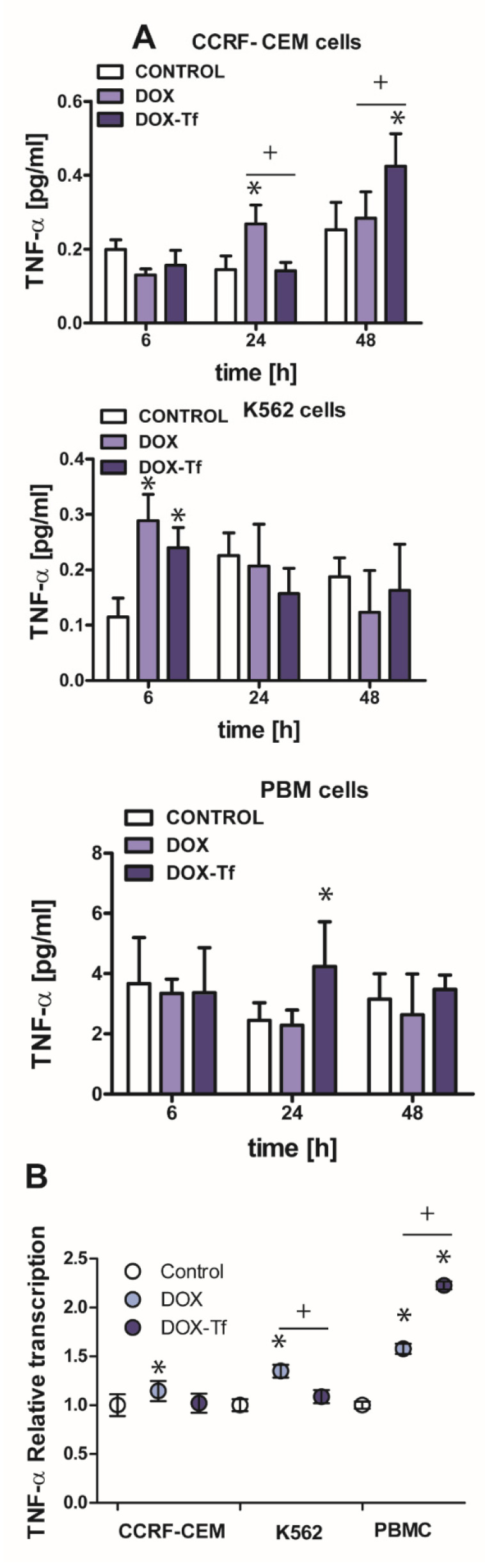
Changes in TNF-α expression in K562 and CCRF-CEM cell lines, and in PBMCs treated with transferrin-bound doxorubicin or free drug (**A**) The effect of IC_50_ concentrations of doxorubicin (DOX) and doxorubicin–transferrin (DOX–Tf) conjugate on TNF-α expression. The data represent mean ± SD of three independent experiments. * *p* < 0.05 in comparison to untreated, control cells, (+) statistically significant differences noted between the probes incubated with free DOX compared to the conjugate, + *p* < 0.05. (**B**) Expression of *TNF-α* gene transcript (relative to *HPRT1* and *HMBS* housekeeping genes) in investigated human leukemia cell lines and PBMCs exposed to IC_50_ concentrations of DOX alone and DOX–Tf conjugate. Asterisks refer to the level of significance (* *p* < 0.05, *n* = 3) whereas symbol “+” indicates a difference between expression in the cells treated with DOX or DOX–Tf, + *p* < 0.05.

In multiple myeloma cells, transcriptional regulation of TRAIL is often triggered by a TNF-α dependent pathway [25]. TNF-α is a multifunctional cytokine capable of inducing several biological responses such as apoptosis, inflammation, and the stress response [26]. Having shown that DOX–Tf conjugate leads to increased mRNA and protein expression of TRAIL, we wished to examine whether TNF-α production is induced by transferrin-bound DOX or free drug. Indeed, the strongest effect of both examined forms of drug was observed in the CCRF-CEM cell line (Figure 5A). These cells displayed more than a 1.5-fold increase in TNF-α production after 24 h incubation with DOX or after 48 h incubation with the conjugate. It is well reported that TNF-α expression can also be regulated at the transcriptional level [27]. By using quantitative real-time RT-PCR, we found that mRNA levels of *TNF-α* increased 1.2-, 1.4- and 1.6-fold following treatment with free DOX in CCRF-CEM, K562, and PBMCs, respectively. However, as shown in Figure 5B, the conjugate-dependent increase in *TNF-α* transcript was observed only in normal PBMCs and showed more than a 2-fold increase in comparison to non-treated cells. 

### 2.6. The Involvement of IL-6 and IL-4 Cytokines in DOX–Tf Cytotoxicity

**Figure 6:**
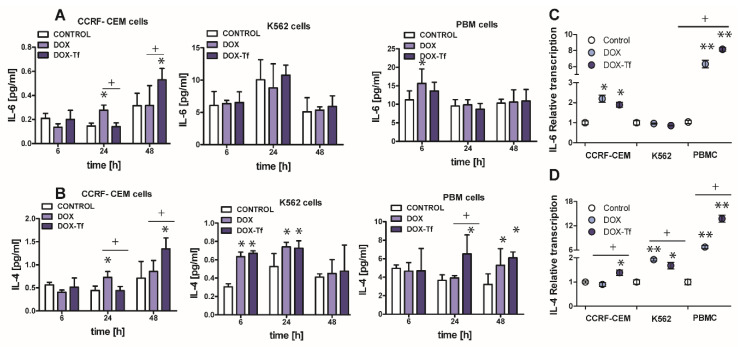
The evaluation of pro-inflammatory cytokines level after leukemia cells treatment with DOX and DOX–Tf conjugate. (**A**,**B**): The concentrations of interleukin 4 (IL-4) and interleukin 6 (IL-6) secreted in the supernatants of CCRF-CEM, K562, and PBMC cultures treated with IC_50_ concentrations of doxorubicin–transferrin (DOX–Tf) conjugate and DOX alone for 6, 24, or 48 h. The results show mean ± SD of three independent experiments. * *p* < 0.05,in comparison to untreated, control cells, (+) statistically significant differences observed between the probes incubated with free DOX in comparison to the conjugate, + *p* < 0.05. (**C**,**D**): Level of mRNA transcripts for *IL-6* and *IL-4* genes in CCRF-CEM, K562, and PBMCs exposed to IC_50_ concentrations of DOX–Tf conjugate and free doxorubicin for 48 h. Data (ΔΔCt values) were transformed into relative copy number values (the number of mRNA copies of the examined genes per housekeeping gene index, calculated as the average Ct value of the *HPRT1* and *HMBS* housekeeping genes) and standardized to the level of mRNA transcripts in untreated cells, taken as 1. Results are presented as the means ± SD, (*n* = 6). Asterisks refer to the level of significant difference (* *p* < 0.05, ** *p* < 0.01) in mRNA level in the drug treated cells compared to untreated cells (control), whereas—symbol “+” (+ *p* < 0.05) refers to statistically significant differences between the cells incubated with DOX–Tf conjugate in comparison to the cells incubated with a reference drug (free DOX).

Apart from TNF–α, another major cytokine that mediates in the induction of apoptosis and the pro-inflammatory response is IL-6 [28]. Normal and malignant human cells secrete these cytokines in response to different types of external stress factors such as radiotherapy, toxins, and chemotherapeutic drugs [29]. It was therefore important to elucidate the role of IL-6 in the toxicity and induction of apoptosis caused by DOX–Tf conjugate. As shown in Figure 6A, the IL-6 expression was up-regulated in PBMC cultures treated with DOX for up to 6 h (1.5-fold increase) and in CCRF-CEM cells incubated with drugs for 24 and 48 h (approximately 2-fold increase with DOX and with the conjugate, respectively). Furthermore, the mRNA level of the gene for *IL-6* was also up-regulated (Figure 6C) in CCRF-CEM cells and in PBMCs treated with either form of DOX. 

**Figure 7:**
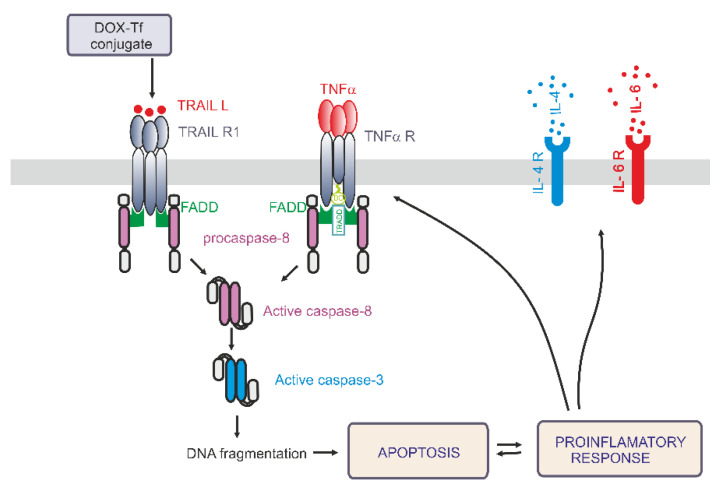
A simplified model of the differential effects of the doxorubicin–transferrin (DOX–Tf) conjugate on the pro-inflammatory response and induction of apoptosis via the extrinsic pathway. DOX–Tf conjugate stimulates the production of TNF-α and TRAIL ligand, the cell signaling proteins. They are also known as two major extrinsic mediators of apoptosis. Acute lymphoblastic cells and chronic myeloid malignancies have different isoreceptors for these cytokines. Binding of TRAIL or TNF-α to their receptors, results in death receptors aggregation (DR4, DR5) and Fas-associated death domain-containing protein (FADD). The death domain of FADD binds to an analogous domain of caspase-8 to form the death-inducing signaling complex (DISC). This process activates downstream caspases and leads to apoptosis. In parallel, in the presence of typical hallmarks of apoptosis, the DOX–Tf conjugate affects cellular genome and mediates the production of pro-inflammatory response. The transcription of IL-4 and IL-6 genes mediates the secretion of these cytokines to the extracellular matrix.

Having ruled out the possibility that IL-6 is always involved in the conjugate-dependent induction of the pro-inflammatory response, we next explored whether IL-4 may participate in the inflammation process that is triggered by these drugs. IL-4 is a cytokine that is produced by T lymphocytes, basophiles, and mastocytes, as well as different types of cancer cells. It was recently reported that IL-4 is crucial during development of leukemia cell sensitivity to chemotherapeutic agents [30]. Figure 6B shows that the conjugate caused a 1.5-fold increase in IL-4 levels in PBMCs after 24 and 48 h of treatment. In human leukemia cell lines, a 1.8-fold and 1.3-fold increase of IL-4 expression was observed in K562 cells after 6 and 24 h incubation with either drug, respectively. In the most sensitive CCRF-CEM cell line, we noticed a time dependence between DOX and conjugate modes of action. Free drug triggered double expression of IL-4 after 24 h treatment, whereas Tf-bound DOX took an additional 24 h to achieve the same effect. A simplified model of the differential effects of DOX–Tf on autophagy is shown in Figure 7.

The data obtained from enzyme-linked immunosorbent assays (ELISA) assays agree with our quantitative RT results (Figure 6D). Therefore, there is an increase in *IL-4* mRNA and protein expression following treatment with either drug in all normal and cancer cell types investigated. Interestingly, there was a predominant IL-4-dependent response of K562 cells to both free DOX and DOX–Tf treatment. This further confirmed the function of IL-4 in the sensitivity of leukemia cells to chemotherapeutic agents.

## 3. Discussion

In the present paper, we address the mechanisms whereby DOX–Tf conjugate kills human leukemia cells in vitro. In particular, we elucidate the involvement of the pro-inflammatory response pathway and its downstream target TRAIL. The main findings and conclusions of the study are summarized in Figure 7. Our results show that the induction of apoptosis via the extrinsic pathway of apoptosis may significantly contribute to the toxicity of the conjugate. Previous studies have demonstrated a potent role of DOX–Tf to cause programmed cell death. Consequently, DOX attached to Tf triggers activation of caspases 3, 8, and 9, decreases mitochondrial membrane potential, produces reactive oxygen species (ROS), and leads to single or dsDNA breaks [21,24]. 

The fatal consequences of DNA damage are often referred to as oligonucleosomal fragmentation, which can be visualized during DNA agarose gel electrophoresis as a ladder-shape pattern. We observed this phenomenon in A549 and HepG2 cells treated with the conjugate for up to 48 h [22]. During apoptosis, the DNA fragments retain free 3’-OH ends, which can be detected via the TUNEL method. It has been previously described that drugs belonging to the first and second generations of anthracyclines induce apoptotic DNA degradation [31]. Moreover, the strong dependence between DNA lesions and apoptosis induced by DOX–Tf conjugate was observed by Lubgan et al [32] in human HL-60-promyelocytic leukemia cells. It was reported that in the multidrug resistant cell line, DOX–Tf induced more dsDNA breaks compared to the free drug. The study also showed that DOX–Tf induced dsDNA breaks more efficiently than free DOX. Furthermore, data described by the same group agreed that the apoptotic rates triggered by DOX–Tf were greater than in the DOX-sensitive parental cell line. Here in our study, we observed that Tf-bound DOX significantly augmented the number of TUNEL-positive cells. Interestingly, the CCRF-CEM cell line, which provides a molecular model of ALL, was the most sensitive to genotoxic DOX–Tf properties with respect to histone H2AX phosphorylation. On the other hand, the K562 cell line, which represents CML, was more resistant to DNA damage induced by either form of DOX compared with CCRF-CEM cells. These data agree with the general evaluation of DOX–Tf conjugate cytotoxicity. Here we show the results obtained after XTT assay readouts, but during earlier experiments we used MTT [33], Neutral red [34], or Alamar Blue [35] assays, which refer to mitochondria, lysosomes, and redox homeostasis, respectively. Even though these assays have different molecular targets, the direction of cellular resistance towards DOX–Tf treatment was the same (K562 < CCRF-CEM < PBMCs). The various sensitivity of the examined human leukemia cells to the conjugate treatment might be related with the TfR expression. The number of TfR per cell is tightly regulated by many different factors such as intracellular iron level, cell proliferation or erythropoiesis at levels of receptor recycling, transcriptional, or post-transcriptional control. Additionally, it was proved that phytohemagglutinin stimulated peripheral blood lymphocytes showed a marked increase in TfR expression a few hours before the initiation of replication [36].

The issue differing sensitivity of normal and cancer cells to the conjugate is more complex and is related to apoptosis induction, DNA damage, TfR expression, and oxidative stress, as well as initiation of local inflammation. Cytokine production has been linked to the pro-inflammatory response, apoptosis, and stimulation of cells towards autophagy [37]. Little is known about cytokines that negatively affect leukemia cell growth and survival. For instance, Pena Martines showed a previously unrecognized role of IL-4 as an inhibitor of the growth and survival of primitive acute myelogenous leukemia (AML) cells. The same group revealed that IL-4 induced apoptosis of AML cells in a Stat6-dependent manner [30]. Therefore, it was interesting to find that the DOX–Tf conjugate-mediated production of IL-4 in both of our leukemic cell lines. Indeed, we noticed that mRNA and protein IL-4 expression was enhanced by free drug and by the conjugate. However, in various types of lymphomas, IL-4 may behave differently. IL-4 increased the sensitivity of diffuse large B-cell lymphoma (DLBCL) subtype to doxorubicin-induced apoptosis and complement-dependent rituximab cell death. In contrast, IL-4 protected ABC-like DLBCL from the cytotoxic effects of doxorubicin and rituximab [38]. The same double-edged sword was demonstrated for IL-6, a cytokine that regulates not only immune and inflammatory responses, but also participates in hepatic acute phase protein synthesis, hematopoiesis, and bone metabolism [39]. IL-6 is known to activate both STAT3 and NF-κB in primary chronic lymphocytic leukemia (CLL) cells. The IL-6 production by CLL B-cells is associated with worse clinical outcome for patients with CLL [40]. Regarding the connection between the cytotoxicity of DOX and IL-6 production, it was found that IL-6 was significantly up-regulated in DOX-treated tissues and cells [41]. We propose that the DOX–Tf-mediated induction of the pro-inflammatory response may be part of a programmed cell death-initiating response (Figure 7). This conclusion agrees with our data showing an increase of IL-6 levels in the supernatant of normal and cancer cells incubated with the conjugate. Furthermore, the overproduction of IL-6 can often appear in parallel to TNF-α secretion [42]. Surprisingly, we observed the same molecular sequence; TNF-α and IL-6 were simultaneously secreted in leukemia cells and in PBMCs following treatment with free DOX or the conjugate. However, TNF-α is not only important in the pro-inflammatory process. As a death ligand, TNF-α can be used for anticancer treatment to induce the extrinsic apoptosis pathway, which is dependent upon TNF-α binding to the death receptor FAS/APO-1, also known as CD95 [43]. The application of antibodies against the CD95 receptor may be a promising therapeutic tool for inducing apoptosis in tumor cells, unless acute and lethal hepatic toxicity appears during cancer treatment [44]. Based on its sequence homology to TNF-α, it has been subsequently identified that TRAIL-L [45] did not affect normal cells and had similar apoptotic effects as TNF-α [46]. However, TRAIL does not always act alone. It was shown that TRAIL in combination with DOX or 4-hydroxy-IFO had highly toxic and pro-apoptotic effects in the TNF-α-sensitive rhabdomyosarcoma cell line KYM-1 [47]. Moreover, DOX can trigger overexpression of *TRAIL-L* mRNA and protein in different types of hepatoma cancer cells [48]. We observed a similar pattern with DOX and DOX–Tf treatment in human leukemia cell lines as well as in normal PBMCs. However, this does not necessarily mean that both forms of DOX trigger pro-death pathways in non-cancer cells. Following the explanation provided by Rogalska et al. [49], we propose that DOX alone, or DOX conjugated to Tf, may activate many effector cells such as cytotoxic T lymphocytes, NK cells, neutrophils, monocytes. or macrophages. Consequently, these drugs might initiate apoptosis through the engagement of death receptors.

In conclusion, we showed that the DOX–Tf conjugate is a novel therapeutic that increases the sensitivity of cancer cell lines and does not affect normal cells. As a promising drug delivery system, Tf-bound DOX induces DNA damage and triggers programmed cell death with the engagement of the TRAIL-dependent, extrinsic pathway of apoptosis. The involvement of TNF-α and other cytokines suggests that pro-inflammatory effects of the conjugate are closely related with its cytotoxicity

## 4. Materials and Methods 

### 4.1. Reagents

DOX was purchased from Sequoia Research Products (Pangbourne, United Kingdom). Transferrin, 2,3-Bis (2-methoxy-4-nitro-5-sulfophenyl)-2*H*-tetrazolium-5-carboxanilide inner salt (XTT) and all reagents for carrying out the conjugation procedure were purchased from Sigma- Aldrich (Darmstadt, Germany). Doxorubicin was bounded to Tf using the modified conjugation procedure developed by Berczi et al. [50], and specified in Patent Claim No WIPO ST 10/C PL 402896. The conjugate was analyzed using spectrofluorometry, mass spectrometry [51], and sodium dodecyl sulfate–polyacrylamide gel electrophoresis (SDS–PAGE), according to Lubgan et al. [32]. TNF-α, TRAIL, IL-6, IL-4, and Human Phospho-Histone H2AX immunoassays were supplied by R&D Systems (Oxford, United Kingdom), whereas the terminal deoxynucleotidyl TUNEL assay was from BioVision (Milpitas, CA, USA). Dulbecco’s Modified Eagle’s Medium (DMEM) and fetal bovine serum (FBS) were supplied by Cambrex (Basel, Switzerland). All other chemicals and solvents used in this study were of the highest analytical grade. 

### 4.2. Cell Culture 

ALL cells (CCRF-CEM cell line, ATCC CCL-119™) were provided by Prof G. Bartosz (Department of Molecular Biophysics, University of Lodz, Lodz, Poland). Myelogenous erythroleukemia cells (K562 cell line, ATCC CCL-243™) were a kind gift from Prof J. Robert at Institute Bergonie, Bordeaux, France. In parallel, the experiments were performed on normal PBMCs. The blood used to isolate these cells was obtained from the Blood Bank in Lodz, Poland. The PBMCs were isolated by centrifugation in a density gradient of Histopaque (300× *g* for 30 min at 22 °C), as described previously [51]. All cells were cultured in RPMI 1640 Cambrex (Basel, Switzerland) containing 2 mM l-glutamine (Invitrogen, Carlsbad, CA, USA), supplemented with 10% FBS (Sigma–Aldrich, Darmstadt, Germany), 100 U/mL penicillin (Invitrogen, Carlsbad, CA, USA), and 100 μg/mL streptomycin (Invitrogen, Carlsbad, CA, USA). The cells were grown in standard conditions (37 °C, 100% humidity, and in an atmosphere containing 5% CO_2_ and 95% normal air). 

### 4.3. Cell Cytotoxicity Assay

The viability of CCRF-CEM, K562, and PBMCs was measured using the XTT cytotoxicity test. The principle of this assay is that viable cells reduce the tetrazolium salt XTT 2,3-bis(2-methyloxy-4-nitro-5-sulfophenyl)-2*H*-tetrazolium-5 carboxanilide to a medium-soluble product (Tunney et al., 2004). Briefly, cells were seeded on 96-well plates at a density of 1 × 10^4^ (CCRF-CEM, K562) or 1 × 10^5^ (PBMC) in each well in 0.1 mL of culture medium. Subsequently, various concentrations of 0.05 mL DOX or DOX–Tf were added to appropriate wells, and cells were treated with drugs for 48h. At the end of the incubation, cells were centrifuged (230× *g* at 4 °C, for 10 min), medium was gently removed, and the cell cultures were resuspended in 50 μL XTT at a final concentration of 0.3 mg/mL medium. The microplates were incubated in a CO_2_ incubator for 4 h and the reduction of XTT was measured at 492 nm using a microtiter plate reader (Awareness Technology Inc., Palm City, FL, USA). The percentage of viable cells was calculated by comparing the reduction of XTT in drug treated cells to non-treated control cells. In parallel, the IC_50_ was quantified (using GraphPad Prism software, Graphpad Inc, San Diego, CA, USA), and this defines the drug concentration causing a 50% reduction of cell viability relative to the control. In parallel with the cytotoxicity experiments, the cellular morphology changes were observed after treatment with free DOX or DOX–Tf by phase contrast microscopy using an IX73 Olympus microscope (Olympus, Tokyo, Japan) equipped with a 20× objective and a Digital Sight camera (Olympus, Tokyo, Japan).

### 4.4. H2AX Assay

The level H2AX protein with phosphorylated Ser139 was measured using a Human Phospho-Histone H2AX DuoSet IC assay kit, according to the manufacturer’s instructions (R&D Systems, Oxford, UK). A total of 2 × 10^6^ cells were incubated for 6 h, 24 h, or 48 h with DOX–Tf or DOX alone. Following incubation, the cells were permeabilized with buffer supplied by the assay manufacturer and incubated with antibodies. Antibodies included primary rabbit monoclonal antibody specific to H2AX (phospho S139; dilution 1:100) and anti-rabbit secondary antibody solution (dilution 1:1000). The fluorescence measurements were obtained on a Fluoroskan Ascent plate reader (Fluoroskan Ascent FL, Stockholm, Sweden) with filter pairs of 540 nm/600 nm and 360 nm/450 nm. The results are presented as the fluorescence ratio measured at 540 nm/600 nm to that measured at 360 nm/450 nm (phosphorylated form to total histone H2AX concentration).

### 4.5. TUNEL Assay

To examine DNA damage associated with apoptosis, a TUNEL assay was used [31]. We performed a detection of the early stages of apoptosis by labeling 3′-OH ends of single- and double-stranded DNA (dsDNA) fragments with bromo-deoxyuridine triphosphate nucleotides (Br-dUTP) according to the Apo-BrdU In Situ DNA Fragmentation Assay Kit protocol supplied by the manufacturer (BioVision Milpitas, CA, USA). Human leukemia cells as well as PBMCs were seeded in 6-well plates at a density of 2 × 10^4^ per well, and were subsequently treated with free DOX or DOX–Tf conjugate for 6 h, 24 h, or 48 h in cell culture growth conditions. After the incubation was complete, cells were collected, washed with; 10 mM phosphate, 0.15 M NaCl, pH 7.4 (PBS), fixed with freshly prepared 4% paraformaldehyde, and incubated for 1 h at 37 °C in DNA Labeling mixture containing TdT Reaction Buffer and terminal deoxynucleotidyl transferase (TdT). Subsequently, the cells were stained with Br-dUTP and anti-BrdU-FITC antibody solution in total darkness for 30 min at room temperature, and then the incubation with propidium iodide/RNase A solution was carried out. Finally, the fluorescence intensity was measured using a Becton Dickinson LSR II flow cytometer (BD Biosciences, Franklin Lakes, N.J., USA) equipped with green and red lasers. The number of TUNEL-positive cells was expressed as a percentage of the total number of cells in the sample.

### 4.6. ELISA

The level of human TRAIL, TNF-α, IL-6, and IL-4 in cell-free culture supernatants was determined in duplicate by ELISAs specific for TRAIL, TNF α, and IL-6 (RnD Systems, Oxford, UK) according to the manufacturer’s recommendation. The absorbance at 450 nm and 560 nm were determined in a microtiter plate reader (Awareness Technology Inc., Palm City, FL, USA). The wavelength values of 450 nm were subtracted from those at 560 nm. Antibody signal intensity was normalized to the total protein amount using the Lowry method. 

### 4.7. Quantitative Real-Time RT-PCR

Total RNA was isolated using the TRI Reagent (Sigma–Aldrich, Darmstadt, Germany), according to the manufacturer’s instructions [22]. Total RNA (5 μg) was used for cDNA synthesis using the Maxima First Strand cDNA Synthesis Kit for RT-qPCR (Thermo Fisher Scientific, Inc., Carlsbad, CA, USA). The real-time PCR analysis was run on a LightCycler 480 SYBR Green I Master Mix (Roche Diagnostics GmbH, Mannheim, Germany) and a Roche LightCycler 480 Instrument (Roche Diagnostics GmbH). The cycling conditions were 95 °C for 1 min, followed by 40 cycles of 95 °C for 5 s, 55 °C for 5 s, and 72 °C for 5 s. Relative gene expression was normalized to the housekeeping genes hydroxymethylbilane synthase (*HMBS*) and hypoxanthine phosphoribosyl transferase (*HPRT*), and was calculated using the ΔΔCt method. The study of mRNA expression included the following genes: *IL-4, IL-6, TNF-α, H2X, and TRAIL-L*. The primer sequences are shown in Table 1.

### 4.8. Statistical Analysis

Statistics were calculated using STATISTICA.PL software v.12.5 (StatSoft, Poland) [33], and the viability curves were prepared using GraphPad Prism 5.0 software (GraphPad Inc., San Diego, CA, USA). All measurements were performed at least in duplicate with *n* = 3–6; a *p* value of 0.05 was considered significant. The data were expressed as the mean ± SD. Statistical significance was evaluated using Student’s t-test or one-way ANOVA followed by Tukey’s test.

## Figures and Tables

**Table 1:** Primer sequences used for RT-PCR.

Gene	Strand	Sequence 5′to 3′
*Hypoxanthine-guanine Phosphoribosyltransferase (HPRT1*)	Forward Reverse	TGACACTGGCAAAACAATGCA GGTCCTTTTCACCAGCAAGCT
*Hydroxymethylbilane synthase (HMBS*)	Forward Reverse	CAAGGACCAGGACATCTTGGAT CCAGACTCCTCCAGTCAGGTACA
*H2AX*	Forward Reverse	GGCCTCCAGTTCCCAGTG TCAGCGGTGAGGTACTCCAG
*TRAIL-L*	Forward Reverse	ACCAACGAGCTGAAGCAGAT CAAGTGCAAGTTGCTCAGGA
*TNF-α*	Forward Reverse	CCCAGGGACCTCTCTCTAATCA GCTACAGGCTTGTCACTCGG
*IL-4*	Forward Reverse	TTGAACAGCCTCACAGAGCAGA GTTGTGTTCTTGGAGGCAGCA
*IL-6*	Forward Reverse	TCTCCACAAGCGCCTTCG CTCAGGGCTGAGATGCCG

## Abbreviations

ALL	acute lymphoblastic leukemia
CML	chronic myelogenous leukemia
DOX	Doxorubicin
DOX–Tf	Doxorubicin–transferrin conjugate
FBS	Fetal bovine serum
*HMBS*	Hydroxymethylbilane synthase (HMBS)
*HPRT1*	Hypoxanthine phosphoribosyltransferase 1
IL-1	interleukin-1
TfR	Transferrin receptors
TNF-α	tumor necrosis factor-α
NF-κB	nuclear factor-κB
TRAIL	TNF-related apoptosis-inducing ligand
FADD	Fas-associated death domain-containing protein
DISC	death-inducing signaling complex
